# HFpEF and Atrial Fibrillation: The Enigmatic Interplay of Dysmetabolism, Biomarkers, and Vascular Endothelial Dysfunction

**DOI:** 10.1155/2022/9539676

**Published:** 2022-10-25

**Authors:** Jure Bosanac, Lara Straus, Marko Novaković, Daniel Košuta, Mojca Božič Mijovski, Jerneja Tasič, Borut Jug

**Affiliations:** ^1^University of Ljubljana, Faculty of Medicine, Ljubljana, Slovenia; ^2^University Medical Centre Ljubljana, Department of Vascular Diseases, Ljubljana, Slovenia

## Abstract

**Background:**

Heart failure with preserved ejection fraction (HFpEF) has a complex pathophysiology that encompasses systemic proinflammatory state and dysregulated levels of cardiometabolic and oxidative stress biomarkers. The prevalence of both HFpEF and atrial fibrillation (AF) is continuously rising, especially in the elderly. The aim of our study was to explore if there were any differences in biomarker levels and vascular function in the elderly patients with HFpEF with and without AF and to assess interconnections between clinically relevant biomarkers and cardiac and vascular function.

**Methods:**

This was a cross-sectional study of patients ≥ 65 years with HFpEF who were divided into 2 groups based on the presence or absence of AF. We have sonographically assessed echocardiographic parameters of left ventricular systolic and diastolic function and the peripheral vascular function parameters, namely, pulse wave velocity (PWV) and flow-mediated dilation (FMD). NT-proBNP, irisin, leptin, adiponectin, insulin-like growth factor 1 (IGF-1), and malondialdehyde (MDA) blood levels were determined.

**Results:**

Fifty-two patients (mean age 80 ± 7 years, 67% females) were included. Patients with HFpEF and AF had significantly lower levels of irisin (median 4.75 vs. 13.5 ng/mL, *p* = 0.007), leptin (median 9.5 vs. 15.0 ng/L, *p* = 0.023), and MDA (median 293 vs. 450 ng/mL, *p* = 0.017) and significantly higher values of NT-proBNP (median 2365 vs. 529 ng/L, *p* < 0.001) but not vascular function parameters, as compared to HFpEF patients without AF. MDA was significantly correlated with diastolic function (*r* = 0.395, *p* = 0.007) and FMD (*r* = 0.394, *p* = 0.011), while adiponectin was inversely associated with FMD (*r* = −0.325, *p* = 0.038) and left ventricular ejection fraction (*r* = −0.319, *p* = 0.029).

**Conclusions:**

Our results have demonstrated that patients with HFpEF and AF have significantly lower leptin, irisin, and MDA levels compared to patients with HFpEF but without AF. These results offer new insights into the complexity of vascular function and cardiometabolic and oxidative stress biomarkers in the context of HFpEF, AF, and aging.

## 1. Introduction

Heart failure with preserved ejection fraction (HFpEF) is emerging as a predominant phenotype of heart failure and is characterized by high burden of noncardiac comorbidities and the lack of effective disease-modifying therapies, which renders medical management challenging and focused mainly on controlling the congestive symptoms and concomitant conditions [1]. The pathophysiology of HFpEF is complex and not yet fully deciphered; however, its associations with noncardiac comorbidities, especially metabolic syndrome, obesity, arterial hypertension, atrial fibrillation (AF), insulin resistance, and chronic kidney disease, have long been acknowledged [2, 3]. Metabolic syndrome likely promotes the stiffening of the myocardium and the diastolic dysfunction, which are both fundamental features of HFpEF. On the other hand, the peripheral metabolic, neurohormonal, and cardiometabolic derangements in HFpEF promote the development of dysmetabolism. In HFpEF patients, these correlations are demonstrated by increased values of metabolic syndrome markers, such as adiponectin and leptin [4–6]. In addition, a newly discovered myokine irisin may hold out similar prospects regarding its potential to serve as a biomarker, as its plasma concentrations are notably elevated primarily in HFpEF. Irisin is produced in the myocardium in response to ischemia, volume overload, inflammation, oxidative stress, and exercise. It plays an essential role in fat metabolism and energy homeostasis and participates in the amelioration of endothelial dysfunction, insulin resistance, and myocardial hypertrophy, which are all common features of HFpEF [7, 8]. Finally, vascular endothelial dysfunction is emerging as a principal hallmark of peripheral derangements in HFpEF and serves as an indicator of dysmetabolic vascular impairment [9, 10]. The comorbidities in HFpEF generate a systemic proinflammatory state which exerts deleterious effects on the myocardium by promoting the adverse cardiac remodeling and on vascular endothelium, thereby decreasing nitrogen oxide bioavailability [11]. Conversely, the endothelial dysfunction itself can have detrimental effects on cardiac metabolism and may promote the development of diastolic dysfunction [12, 13]. Heart failure and AF are common cardiovascular disorders that frequently occur simultaneously and can exert important adverse effects on each other and on overall cardiovascular health. The prevalence of both conditions is continuously increasing as the risk factors underlying each condition are becoming more common. In fact, AF is the most common sustained cardiac arrhythmia and affects from 1.5 to 2% of the population in the developed countries. Because the risk for developing AF steeply rises after the age of 65, the prevalence and socioeconomic burden of AF are expected to increase significantly in the following 50 years due to the aging population [14, 15]. There are several mechanisms by which heart failure can cause AF, including atrial pressure overload and enlargement, altered myocardial electrical conduction, and structural remodeling of the atria, which create a vulnerable proarrhythmic environment that contributes to the initiation and maintenance of AF. Conversely, AF can lead to the decompensation of heart failure or it can be the primary initiating factor for its development [16]. Interestingly, a study published in 2019 has proposed that the incidence of AF among people older than 80 years old reaches up to 17%.

The exact pathophysiological connection of AF and advanced age itself is not yet completely understood. It has been suggested that aging causes changes in the heart collagen matrix, which consequently induces atrial fibrosis that can lead to the development of AF. Additionally to the impact of the advanced age on the development of AF, the elderly are more often predisposed to various comorbidities, such as ischemic heart disease and heart failure, which could play their role in the pathogenesis of AF. Besides the anatomical substrate, the aging population was found to be more prone to an increased p-wave duration, which precedes the onset of AF [17, 18]. There are still significant gaps in the scientific understanding of the pathophysiology of AF in patients with HFpEF. Indeed, the interrelations among the coexisting comorbidities, which commonly accompany HFpEF, inevitable process of aging, HFpEF itself, and AF, have been insufficiently studied and remain largely undetermined. The biomarkers of cardiometabolic dysregulation and oxidative stress are particularly understudied in this population. Furthermore, the complex interactions between metabolic derangements, cardiac dysmetabolism, biomarkers, endothelial dysfunction in the context of HFpEF, and AF remain elusive and incompletely pathophysiologically and clinically elaborated to this day, especially in the elderly. The associations of all the aforementioned phenomena with AF in the elderly with HFpEF have not been examined so far.

The aim of our study was to investigate if cardiometabolic biomarkers, oxidative stress biomarkers, and vascular and diastolic function are (a) different in a patient subgroup with AF when compared with a subgroup without AF and (b) if they are interrelated with each other in elderly patients with HFpEF.

## 2. Methods

### 2.1. General Data

This was a cross-sectional study of the 52 consecutive patients with HFpEF of hypertensive, valvular, or ischemic etiology or a combination thereof, who have visited the Department of Vascular Diseases Cardiology Outpatient Clinic at the University Medical Centre in Ljubljana between May 2021 and February 2022. Patients underwent a thorough clinical cardiovascular assessment, including the vascular assessment and biomarker appraisal. In 5 patients, we were unable to obtain plasma concentrations of IGF-1, adiponectin, irisin, leptin, and malondialdehyde (MDA). Furthermore, we were unable to assess PWV in 5 patients and FMD in 2 patients. Additionally, we have acquired incomplete measurements of PWV in 1 patient and FMD in 3 patients. Complete echocardiography was performed in the period within 6 months before the visit. We have included patients equal to or older than 65 years of age with HFpEF. HFpEF was diagnosed as the presence of symptoms and signs of heart failure with left ventricular ejection fraction (LVEF) more or equal to 50% and the objective evidence of structural and/or functional cardiac abnormalities consistent with the presence of left ventricular diastolic dysfunction, including the LA volume index (LAVI) greater than 34 mL/m^2^, NT-proBNP levels above 125 pg/mL, E/e′ ratio over 9, and systolic pulmonary artery pressure (sPAP) greater than 35 mmHg [1]. Exclusion criteria included acute illness 1 month prior to inclusion (cardiac decompensation requiring hospital admission, unplanned specialist management, or emergency visit), paroxysmal or persistent AF, other unstable dysrhythmias, and intellectual disability, including dementia. Written consent was obtained from all patients prior to their inclusion. The study was approved by the National Medical Ethics Committee.

### 2.2. Vascular Assessment

Flow-mediated dilation (FMD) was determined with Aloka ProSound *α*7 ultrasound with a 10 MHz linear probe. Before each measurement, a pneumatic cuff was placed on a supine patient's antebrachium and inflated to 20 mmHg above the systolic pressure [12]. After 4.5 minutes, the circulation was restored, and the brachial artery was scanned in longitudinal view approximately 3 cm above the cubital fossa. Images were obtained simultaneously with the ECG tracing and digitally recorded in late diastole [12]. Maximal dilation of the brachial artery was recorded 60 seconds after the cuff deflation, and the averages of 3 measurements of the baseline and posthyperemic diameters were used for analysis [13]. FMD was expressed as the percentage change of brachial artery diameter from the baseline value to the maximum increase during hyperemia [12, 19].

The assessment of arterial stiffness was performed with the same ultrasound by using an echo tracking program and 10 MHz linear probe. The patients were placed supine with their heads resting slightly elevated above the bed and leaned toward their left side. The probe was then positioned above the anterior wall of the right common carotid artery, which was scanned in the longitudinal axis around 1.5 to 2 cm inferior to the bifurcation. The cursor pair on the ultrasound screen was positioned on the anterior and posterior wall of the common carotid artery. To accomplish the calibration, the arterial blood pressure was measured on the right brachium before the first and after the sixth image acquisition. The program has performed an automatic analysis of 12 consecutive pulse pressure waveforms and calculated *β*-stiffness index and pulse wave velocity (PWV) as an average [20].

### 2.3. Biomarker Appraisal

Blood was collected from the antecubital vein according to the standard procedure and collected into two vacuum tubes. The first tube contained 0.11 mol/L sodium citrate, and the second contained a coagulation activator and separating gel. Plasma and serum were prepared by centrifugation at 2500 × g for 20 minutes, aliquoted into plastic vials, snap frozen in liquid nitrogen, and stored at ≤ -70°C. Plasma concentrations of IGF-1 (human IGF-I/IGF-1 Quantikine ELISA Kit, Cusabio) and MDA (MDA (malondialdehyde) ELISA Kit, Elabscience) were measured using a classical sandwich ELISA. Serum irisin concentration was measured with a classical sandwich ELISA (human irisin ELISA kit, Cusabio), whereas concentrations of adiponectin and leptin were determined with xMAP technology on a MAGPIX analyzer (R&D Systems) according to the manufacturer's instructions.

### 2.4. Statistical Analysis

The distribution of variables was assessed using the Kolmogorov-Smirnov test after the previous graphical description. Continuous variables were expressed as mean values and standard deviations if normally distributed or as median and interquartile ranges, if asymmetrically distributed. Categorical variables were expressed as numbers and percentages. Independent samples *t*-test and Mann–Whitney *U* tests were performed for comparison of two groups for normally and asymmetrically distributed variables, respectively. The chi-square test was used to assess differences of categorical variables. The associations between 2 variables were determined using Spearman's correlation coefficient. Data was analyzed with the IBM SPSS Statistics v. 20. A value of *p* < 0.05 was considered statistically significant.

## 3. Results

52 patients were included in our study, and 67% of them (*n* = 35) were females. The average age of patients was 80.6 years (Table 1). Patients were divided into two groups based on the presence or absence of AF. There were no significant differences between groups in terms of demographic data, cardiovascular risk factors, and obtained echocardiographic parameters. However, patients with AF had significantly lower levels of irisin (median 4.75 vs. 13.5 ng/mL, *p* = 0.007), leptin (median 9.5 vs. 15.0 ng/L, *p* = 0.023), and MDA (median 293 vs. 450 ng/mL, *p* = 0.017) and significantly higher values of NT-proBNP (median 2365 vs. 529 ng/L, *p* < 0.001) as compared to patients without AF (Table 1). Differences in adiponectin and IGF-1 levels were not significantly different between the two groups. Also, no significant differences between groups were seen in terms of FMD and PWV (Table 1). In the AF group, half of the patients were taking direct oral anticoagulants, while 40% of patients were taking vitamin K antagonists. Because of the major bleeding episodes in the past, 10% of patients were not taking any anticoagulant therapy.

We have assessed the associations between biomarkers, echocardiographic parameters, and markers of vascular function. FMD was significantly associated with MDA (*r* = 0.394, *p* = 0.011), while PWV was inversely associated with adiponectin levels (*r* = −0.325, *p* = 0.038). Adiponectin was also inversely correlated with LV EF (*r* = −0.319, *p* = 0.029), while MDA was significantly associated with E/e′ (*r* = 0.395, *p* = 0.007). NT-proBNP, which is an established biomarker of heart failure, was significantly inversely associated with irisin (*r* = −0.420, *p* = 0.003) and leptin levels (*r* = −0.354, *p* = 0.015) (Table 2).

## 4. Discussion

In our study, we have demonstrated that older patients with HFpEF and AF had lower plasma levels of cardiometabolic (irisin, leptin) and oxidative stress (malondialdehyde) biomarkers, as compared to those without AF. Additionally, levels of some of these biomarkers significantly correlate with parameters of vascular function and echocardiographic features of heart failure worsening. Our study is the first to assess these interrelations in a rapidly growing population of the elderly with HFpEF.

We have shown that irisin levels are significantly lower in patients with HFpEF with AF as compared to HFpEF without AF. There may be several possible pathophysiological explanations for these results. Firstly, the cardiac electrical conduction depends on stable cardiac electrophysiology, normal cardiac structure, and a balanced input to the heart from an autonomic nervous system. Calcium ions play a central role in cardiomyocyte membrane potential regulation and are essential for the repolarization of the cells and the conduction of action potential. A recent preclinical study has found that irisin treatment significantly increases intracellular calcium concentration via an irisin-specific membrane receptor in rat cardiac cells. Moreover, irisin's precursor, FNDC5, activates the pathway in the hypothalamic paraventricular nucleus, which then decreases the circulating levels of noradrenaline and subsequently inhibits the sympathetic tone overdrive [21]. Altered calcium ion homeostasis in the state of low plasma irisin level may contribute to atrial arrhythmogenesis through impaired atrial refractoriness, proarrhythmic delayed afterdepolarizations, and cardiac autonomic dysregulation. Moreover, the association of AF with the lower plasma concentrations of irisin may be due to the absence of irisin's inhibitory action on the sympathetic nervous system. Consequently, the imbalance in sympathetic tone to the heart creates a transitory dynamic proarrhythmogenic substrate which may trigger and initiate the AF [22–24].

Secondly, the association between irisin levels and AF in patients with HFpEF may be explained by cardiomyocyte fibrosis, which is in turn associated with the occurrence of AF. It was demonstrated that increased FNDC5 and irisin expression prevents the impairment of autophagy and lipid accumulation in the myocardium and mitigates the detrimental cardiac remodeling. Additionally, an increased expression of FNDC5 and irisin alleviates the obesity-induced cardiac inflammation, oxidative stress, and hypertrophic remodeling of the heart. What is more, irisin reduces the effects of lipotoxicity, mitigates cardiomyocyte apoptosis, alleviates myocardial inflammation, and decreases oxidative damage. Studies suggest that irisin acts by suppressing the activity of cardiac fibroblasts, which subsequently inhibits the collagen synthesis, impedes myofibroblast activation, and inhibits the fibrotic transformation of the myocardium. Moreover, irisin promotes angiogenesis and thus even further reduces cardiac fibrosis [21]. In conclusion, the lack of the listed protective effects of irisin could explain the occurrence of AF—by mechanisms of enhanced atrial fibrosis, adverse remodeling, increased inflammation, and oxidative stress.

Similarly, we have shown that leptin levels were significantly lower in patients with HFpEF and AF as compared to HFpEF without AF [24]. The mechanisms by which leptin impacts atrial electrophysiology remain incompletely understood. Literature regarding the association of blood leptin levels and heart rate variability parameters indicates a possible link between leptin concentration and the disturbances of the autonomic nervous system in some ethnicities. One study reported that hyperleptinemia might be directly associated with cardiac autonomic dysfunction in patients with type 2 diabetes mellitus and visceral obesity, which is not in line with our results. The reason for such discrepancy may lay in patients' age, level of obesity, and level of atrial fibrosis. Our results may suggest a new and yet unexplained mechanism for such phenomenon and require future studies to examine it [25].

The observed inverse correlation between the flow-mediated dilation (FMD) measurements and adiponectin plasma concentrations in our study seems contradictory to the established facts about adiponectin's effects on vascular function. The enhanced endothelial function expressed with the higher values of FMD cannot be explained by the lack of adiponectin's supposedly beneficial and protective effects on nitric oxide bioavailability and endothelial function, which was shown in most literature reports. Nevertheless, one study might offer a potential explanation for an inverse correlation between adiponectin levels and FMD. It has demonstrated that adiponectin and lectin-like oxidized LDL receptor 1 have exhibited a reciprocal pattern in the state of inflammation. Similarly, studies in type 2 diabetes mellitus in mice have presented a reciprocal regulation between adiponectin and tumor necrosis factor *α* affecting the regulation of both coronary and aortic endothelial functions. Therefore, lower concentrations of adiponectin in the state of normal endothelial function might be explained by the systemic inflammatory state which occurs in patients with HFpEF and may not as readily affect the endothelial function as it does the reduction of adiponectin concentration, as it is suggested by the reciprocal pattern [26]. Given the aforementioned findings, we may conclude that adiponectin plays an important role in vascular signaling and in the regulation of endothelial vascular function; however, despite the strong experimental data regarding the advantageous effects of adiponectin on endothelial function, some clinical studies, including ours, have yielded contradictory results. For example, adiponectin has been associated with either reduced or increased cardiovascular risk in different studies, which may indicate the complex and not yet completely understood mechanisms by which adiponectin acts on the cardiovascular system. Moreover, adiponectin synthesis and plasma levels are also dependent on the underlying diseases and metabolic state as well, thus adding to the complexity of assessing its plasma levels. Additionally, adiponectin expression is suppressed in obesity, while the development of heart failure is associated with a significant increase in adiponectin plasma levels. Therefore, to elucidate the effect of adiponectin more conclusively, the secretory profile of adiponectin may need to be considered in the broader context of the comorbidities of HFpEF [27].

Since malondialdehyde is a marker of lipid peroxidation and PWV reflects stiffness of the arteries, these two parameters can be interconnected. A study discovered that PWV and plasma concentration of MDA are positively associated in individuals with metabolic syndrome in comparison to individuals without metabolic syndrome. Hyperglycemia causes an increased activity of the renin-angiotensin-aldosterone system on the local level, and it is well acknowledged that aldosterone causes proliferation of the smooth muscle and increases collagen synthesis in the vessel wall. The exact function of MDA regarding the increase in stiffness of arterial wall is still unknown, but it is assumed that the enhanced production of reactive oxygen species in the vascular wall in the state of comorbidities of HFpEF can lead to adverse remodeling of the vascular wall, which increases its stiffness and the measured values of PWV [28]. Our results support this hypothesis. To conclude, our findings are consistent with the already accepted theories regarding the effects of oxidative stress on the deleterious remodeling of the arterial walls that leads to the higher values of PWV. However, our another result which shows that MDA levels are significantly lower in patients with HFpEF with AF as compared to HFpEF without AF is not in line with most literature reports. Furthermore, in our extensive literature search, we were unable to obtain any data that would offer explanation to this finding. Thereby, this association warrants future research. As for the association between MDA and diastolic function, to the best of our knowledge, this is the first study to assess these associations in the elderly with HFpEF. MDA is considered a marker of oxidative stress [29] in patients with heart failure with reduced ejection fraction and was shown to be a significant predictor of worse outcomes and mortality in these patients [30, 31]. We have demonstrated that MDA is associated with left ventricular filling pressure, a proxy of diastolic dysfunction in HFpEF, which is a significant predictor of worse outcome in these patients. Studies have shown that cardiac relaxation is associated with oxidative stress, probably as a consequence of proinflammatory state in the endothelium that further leads to sarcomere stiffness in the myocardium [32]. Additionally, an interesting but expected finding is therefore a significant association between MDA and PWV, which suggests that arterial stiffening in the elderly is a dynamic process and is associated with the oxidative stress. Furthermore, in a study of Lambadiari and colleagues, a decrease in MDA levels was significantly correlated with PWV decrease, confirming their interrelation [33].

Although most of our results are novel in comparison with previous literature reports, we have identified some limitations in our study. The first limitation is a sample size and a single-center setting. Although the HFpEF population is rapidly growing, very few studies were performed in the elderly population only, which is an important strength of our study. Secondly, it is a cross-sectional study and can therefore answer only the questions of association and not of causality. Thirdly, we have included only patients with permanent AF, while patients with other types (e.g., paroxysmal or persistent) have been excluded, which lowers the generalizability of our results. Fourthly, the missing data can reduce statistical power and produce biased estimates, thus leading to incorrect conclusions.

## 5. Conclusion

Nevertheless, our results provide new insights into the complexity of vascular function and cardiometabolic and oxidative stress biomarkers in the context of HFpEF, AF, and aging. According to our results, older patients with HFpEF and AF have significantly lower leptin, irisin, and MDA levels compared to the older patients with HFpEF but without AF. Finally, we have shown that oxidative stress is associated not only with vascular function but also with diastolic cardiac function. Future studies are warranted to address the missing steps in the pathophysiological cascade in this rapidly growing population of elderly with HFpEF.

## Figures and Tables

**Table 1:** Demographics, clinical data, biomarkers, and parameters of vascular and cardiac function.

	All	HFpEF with AF	HFpEF without AF	*p*
Age, mean (SD), and years	80.6 (6.6)	81.6 (6.9)	79.3 (6.0)	0.216
Female sex, *n* (%)	35 (67%)	17 (57%)	18 (82%)	0.076
BMI, mean (SD) (kg/m^2^)	31.3 (7.1)	31.1 (8.5)	31.2 (4.9)	0.779
NYHA I/II/III, *n*	7/40/5	3/24/3	4/16/2	0.694
Coronary heart disease, *n* (%)	14 (27%)	8 (27%)	6 (27%)	0.961
Valvular heart disease, *n* (%)	10 (19%)	7 (23%)	3 (14%)	0.381
Arterial hypertension, *n* (%)	51 (98%)	30 (100%)	21 (96%)	0.238
Smoking, *n* (%)	19 (37%)	11 (37%)	8 (36%)	1.000
Diabetes mellitus, *n* (%)	17 (33%)	7 (23%)	10 (45%)	0.093
LV EF, mean (SD) (%)	63.2 (6.4)	63.3 (5.7)	63.1 (7.3)	0.945
E/e′, mean (SD)	14.2 (4.1)	14.2 (4.1)	14.2 (4.1)	0.990
LAVI, mean (SD) (mL/m^2^)	46.0 (15.8)	52.1 (16.7)	37.6 (9.7)	**<0.001**
sPAP, mean (SD) (mmHg)	37.9 (11.3)	40.2 (12.7)	34.8 (8.5)	0.072
Mitral regurgitation no/mild/moderate, *n*	6/39/7	2/23/5	4/16/2	0.363
ACEi/ARB percentage of maximal dose, median (Q1-Q3) (%)	50 (16-100)	50 (10-100)	50 (19-100)	0.657
Beta blockers percentage of maximal dose, median (Q1-Q3) (%)	25 (25-69)	50 (25-100)	25 (13-50)	0.069
Loop diuretics, *n* (%)	37 (71%)	24 (80%)	13 (59%)	0.100
FMD, mean (SD) (%)	9.7 (6.5)	9.4 (6.7)	10.0 (6.4)	0.792
PWV, mean (SD) (m/s)	8.0 (1.3)	7.8 (1.4)	8.4 (1.2)	0.122
NT-proBNP, median (Q1-Q3) (ng/L)	1280 (535-2585)	2365 (1310-4123)	529 (228-732)	**<0.001**
IGF-1, median (Q1-Q3) (ng/mL)	22.3 (17.0-37.3)	25.2 (16.8-44.2)	22.3 (17.0-36.1)	0.871
Irisin, median (Q1-Q3) (ng/mL)	7.7 (3.5-19.5)	4.8 (2.6-12.7)	13.5 (7.1-31.5)	**0.007**
Leptin, median (Q1-Q3) (ng/L)	11.0 (6.0-18.0)	9.5 (5.3-15.0)	15.0 (11.0-26.0)	**0.023**
Adiponectin, median (Q1-Q3) (ng/L)	456 (336-491)	420 (334-494)	472 (398-491)	0.474
MDA, median (Q1-Q3) (ng/mL)	352 (283-580)	293 (273-423)	450 (342-952)	**0.017**

HFpEF: heart failure with preserved ejection fraction; AF: atrial fibrillation; SD: standard deviation; Q1-Q3: interquartile range; BMI: body mass index; NYHA: New York Heart Association; LVEF: left ventricular ejection fraction; E/e′: E-wave divided by e′ velocity; LAVI: left atrial volume index; sPAP: systolic pulmonary pressure; ACEi/ARB: angiotensin-converting enzyme inhibitors/angiotensin receptor blockers; FMD: flow-mediated dilation; PWV: pulse wave velocity; NT-proBNP: N-terminal probrain natriuretic peptide; IGF-1: insulin-like growth factor 1; MDA: malondialdehyde.

**Table 2:** Association between examined biomarkers with cardiac and vascular function. The numbers in cells represent Spearman's correlation coefficients. Statistically significant associations (*p* <0.05) are marked in italics.

	FMD	PWV	LV EF	E/e′	LAVI	sPAP	NT-proBNP
IGF-1	-0.168	-0.121	0.076	0.235	-0.003	-0.217	-0.201
Irisin	-0.193	0.194	-0.150	0.031	-0.229	-0.160	*-0.420*
Leptin	-0.227	0.182	-0.105	-0.070	-0.221	-0.182	-*0.354*
Adiponectin	*-0.368*	0.017	*-0.319*	-0.069	0.057	0.014	-0.178
MDA	-0.166	*0.382*	0.057	*0.352*	-0.231	-0.019	-0.075

IGF-1: insulin-like growth factor 1; MDA: malondialdehyde; FMD: flow-mediated dilation; PWV: pulse wave velocity; LVEF: left ventricular ejection fraction; E/e′: E-wave divided by e′ velocity; LAVI: left atrial volume index; sPAP: systolic pulmonary pressure; NT-proBNP: N-terminal probrain natriuretic peptide.

## Data Availability

The data used to support the findings of the study are available from the corresponding author upon request.
